# *Thonningia* *sanguinea* Extract: Antioxidant and Cytotoxic Activities Supported by Chemical Composition and Molecular Docking Simulations

**DOI:** 10.3390/plants10102156

**Published:** 2021-10-11

**Authors:** Reda F. A. Abdelhameed, Sameh S. Elhady, Alaa Sirwi, Hanan Samir, Elsayed A. Ibrahim, Ama Kyeraa Thomford, Alaa El Gindy, Ghada M. Hadad, Jihan M. Badr, Mohamed S. Nafie

**Affiliations:** 1 Department of Pharmacognosy, Faculty of Pharmacy, Suez Canal University, Ismailia 41522, Egypt; reda.abdelhameed@pharm.suez.edu.eg; 2 Department of Natural Products, Faculty of Pharmacy, King Abdulaziz University, Jeddah 21589, Saudi Arabia; ssahmed@kau.edu.sa (S.S.E.); asirwi@kau.edu.sa (A.S.); 3 Department of Pharmaceutical Analytical Chemistry, Faculty of Pharmacy, Suez Canal University, Ismailia 41522, Egypt; hanan.samir@pharm.suez.edu.eg (H.S.); elsayed_ibrahim@pharm.suez.edu.eg (E.A.I.); alaaeldeen_mohamed@pharm.suez.edu.eg (A.E.G.); ghada_tawfeek@pharm.suez.edu.eg (G.M.H.); 4 Medical Administration, Student’s Hospital, Zagazig University, Zagazig 44519, Egypt; 5 Department of Biomedical Sciences, School of Allied Health Sciences, University of Cape Coast, Cape Coast PMB TF0494, Ghana; ama.thomford@ucc.edu.gh; 6 Department of Chemistry, Faculty of Science, Suez Canal University, Ismailia 41522, Egypt; mohamed_nafie@science.suez.edu.eg

**Keywords:** *Thonningia* *sanguinea*, antioxidant, cytotoxic, LC-ESI-TOF-MS/MS, molecular docking

## Abstract

The current study was designed to investigate the antioxidant and cytotoxic activities of *Thonningia sanguinea* whole-plant extract. The total phenolic content was determined using Folin–Ciocalteu reagent and found to be 980.1 mg/g, calculated as gallic acid equivalents. The antioxidant capacity was estimated for the crude extract and the phenolic portion of *T. sanguinea*, whereupon both revealed a dose-dependent scavenging rate of DPPH^•^ with EC_50_ values of 36.33 and 11.14 µg/mL, respectively. Chemical profiling of the plant extract was achieved by LC-ESI-TOF-MS/MS analysis, where 17 compounds were assigned, including ten compounds detected in the negative mode and seven detected in the positive mode. The phenolic portion exhibited promising cytotoxic activity against MCF-7 and HepG2 cells, with IC_50_ values of 16.67 and 13.51 μg/mL, respectively. Phenolic extract treatment caused apoptosis in MCF-7 cells, with total apoptotic cell death 18.45-fold higher compared to untreated controls, arresting the cell cycle at G2/M by increasing the G2 population by 39.7%, compared to 19.35% for the control. The apoptotic investigation was further validated by the upregulation of proapoptotic genes of P53, Bax, and caspases-3,8 9, and the downregulation of Bcl-2 as the anti-apoptotic gene. Bcl-2 inhibition was also virtualized by good binding interactions through a molecular docking study. Taken together, phenolic extract exhibited promising cytotoxic activity in MCF-7 cells through apoptosis induction and antioxidant activation, so further fractionation studies are recommended for the phenolic extract for specifying the most active compound to be developed as a novel anti-cancer agent.

## 1. Introduction

Cancer is a significant cause of death around the world [1,2]. Because of their independence from normal regulatory signals, cancer cells develop into tumor tissues, which can then move to other organs in a process known as metastasis, which is responsible for approximately 90% of cancer-related fatalities [3,4,5]. As a result, developing novel chemotherapeutic drugs capable of being used in cancer therapy is critical. As healthcare becomes less paternalistic and the demand for more candidate drugs grows more critical, plant-based health systems will continue to play an essential role in healthcare, and their use by various cultures will change and increase [6,7,8]. Medicinal plants have made a significant contribution to the rapidly expanding discipline of natural product chemistry. Natural products have also been accepted as a significant source in satisfying the healthcare demands of many societies, as a result of several discoveries [9]. Hence, natural products are part of current and effective tools for developing novel pharmaceuticals, as well as a bank of pharmacological templates [10,11,12,13]. Medicinal plants possess a significant impact in the following areas: antimicrobial [14], anti-inflammatory [15], anthelminthic [16,17], antidiabetic [18], and anticancer activity [19,20].

In a continuing effort to discover biologically active phytochemicals from medicinal plants [21,22,23,24,25], our group focused on the study of *T. sanguinea*, a locally well-known medicinal plant widely distributed throughout the forest zones of tropical Africa. *T. sanguine* is rich in phenolic compounds, such as brevifolin carboxylic acid and gallic acid [26]. In addition, dihydrochalcone glucoside derivatives, coniferin, (+)-pinoresinol, and lariciresinol were reported [27,28]. The compounds isolated from this plant are well known for their interesting biological activities. A previous survey was conducted and declared its use in folk medicine to treat coughs, urinary tract infections, and erectile dysfunction, and as an anthelmintic [29]. Additionally, it is also used for the treatment of sore throat, skin infections, abscesses, dental caries, gingivitis, fever, malaria, heart disease, and rheumatism [30,31]. Previous studies proved antimicrobial and antifungal effects, alongside the hepatoprotective activities of the *T. sanguinea* extracts [27,28]. Additionally, Ayim and Bayor et al. evaluated the cytotoxic activity of a selected number of Ghanaian plants. Among the investigated plants were *T. sanguinea* roots, where its activity was studied against three human cancer cell lines, DLD-1 (colon), MCF-7 (breast), and M14 (melanoma). The study revealed moderate IC_50_ values of 40 ± 1.0, 55 ±1.2, and 43.2 ± 2.0, respectively [32]. These findings prompted us to investigate the whole plant in terms of chemical composition using LC-ESI-TOF-MS/MS analysis, the total phenolic contents, and the antioxidant activity of *T. sanguinea*. The plant extract was found to inhibit the proliferation of human hepatocellular carcinoma cells (HepG-2) through apoptosis induction. This was elucidated by cell cycle arrest in the sub-G1 cell population and DNA fragmentation [33]. Therefore, the current study was planned to screen the cytotoxic activity of crude and phenolic extract of *T. sanguinea* against another panel of cancer cell lines, and investigate the apoptosis induction in the tested cell line. Furthermore, the scope of this paper was extended for exploring the virtual mechanism of binding of the identified compounds through molecular docking towards the Bcl-2 protein.

## 2. Results and Discussion

### 2.1. Total Phenolic Content

The total phenolic content was determined in the extract using Folin–Ciocalteu reagent by spectrophotometric technique, and was found to be 980.1 mg/g, calculated as gallic acid equivalents.

### 2.2. Biological Activities

#### 2.2.1. Antioxidant Activity

**Figure 1:**
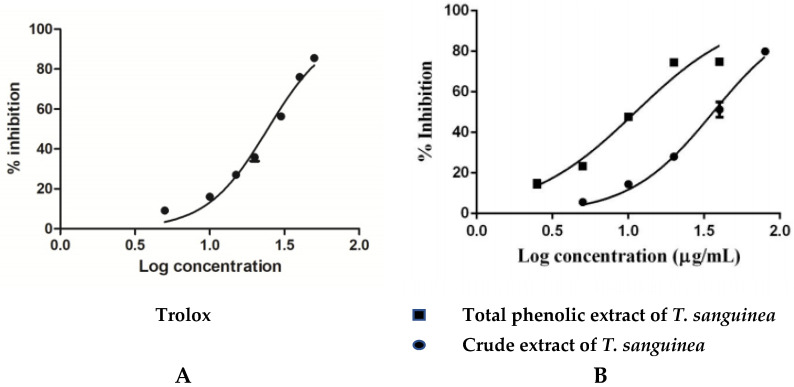
Chemical DPPH^•^ scavenging rate by Trolox (**A**), *T*. *sanguinea* crude and phenolic extracts (**B**). All values are expressed as means (%) and SE of triplicated experiments.

The antioxidant activity was estimated for the crude extract and the phenolic portion of *T*. *sanguinea* using DPPH^•^ reagent. As shown in Figure 1, both the crude extract and the phenolic portion revealed a dose-dependent scavenging rate of DPPH^•^. *T*. *sanguinea* crude extract revealed EC_50_ values of 36.33 ± 1.02 µg/mL, and the phenolic portion exhibited EC_50_ values of 11.14 ± 1.06 µg/mL compared with the EC_50_ (24.42 ± 0.87 µg/mL) that was shown by the positive control, Trolox. The exhibited scavenging rate of the measured samples, as well as the used positive control, are illustrated in Figure 1. These results agree with the previously published work [23] that suggests *T*. *sanguinea* with radical scavenging action might ameliorate AFB1-induced liver toxicity.

#### 2.2.2. Cytotoxic Activity

**Figure 2:**
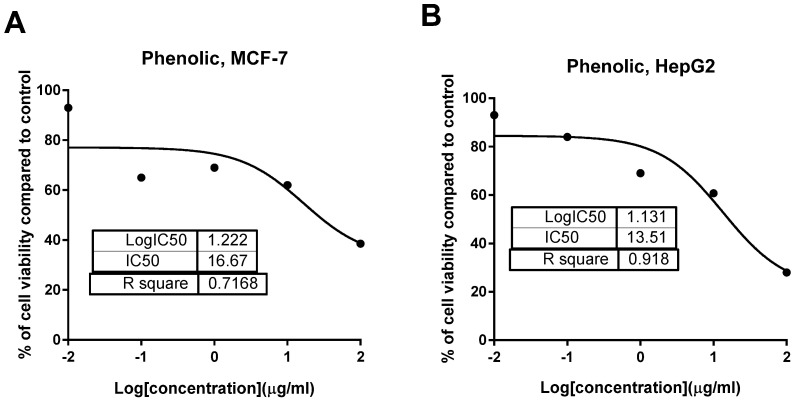
Dose–response nonlinear regression curve fitting the percentage of cell viability vs. log [con. µg/mL], R square ≈1, using the GraphPad prism software. (**A**) Cytotoxicity of phenolic extract against MCF-7; (**B**) cytotoxicity of phenolic extract against HepG2 cell line.

Previous investigation of *T. sanguinea* roots for its cytotoxic activity against three human cancer cell lines, DLD-1 (colon), MCF-7 (breast), and M14 (melanoma), revealed moderate IC_50_ values of 40, 55, and 43.2, respectively [10]. In another previous study on *T. sanguinea* subaerial extract, the isolation of ten dihydrochalcone glucoside derivatives was reported. Namely, they were: 3-hydroxyphloridzin, 2′-*O*-(6-*O*-Galloyl-*β*-d-glucopyranosyl)-3-hydroxyphloretin, 2′-*O*-(4,6-*O*-Sa-Hexahydroxydiphenoyl-*β*-d-glucopyranosyl)-3-hydroxyphloretin, 2′-*O*-(3-*O*-Galloyl-4,6-*O*-Sa hexahydroxydiphenoyl-*β*-d-glucopyranosyl)- 3-hydroxyphloretin, 4′-*O*-(4,6-*O*-Sa-Hexahydroxydiphenoyl-*β*-d-glucopyranosyl)- phloretin, 2′-*O*-(3-*O*-Galloyl-4,6-*O*-Sa-hexahydroxydiphenoyl-*β*-d-glucopyranosyl) phloretin, 2′,6′- dihydroxy-4′-(*β*-d-glucopyranosyloxy) dihydrochalcone, 2′,6′-Dihydroxy-4′-*O*-(6-*O*-galloyl-*β*-d-glucopyranosyl)- dihydrochalcone, thonningianin B, and thonningianin A. In addition, coniferin, (+)-pinoresinol, and lariciresinol were reported [27]. In our present study, both crude and phenolic extract of the whole-plant *T. sanguinea* were tested for their cytotoxic activity using an MTT assay. As seen in Table 1 and Figure 2, phenolic extract exhibited promising cytotoxic activity against MCF-7 and HepG2 cells with IC_50_ values of 16.67 and 13.51 μg/mL, respectively. These promising activities were compared to 5-FU of higher IC_50_ values of 26.98 and 15.8 μg/mL, respectively. At the same time, it did not have any detected activity with IC_50_ values higher than 50. This might indicate their selectivity of action. On the other hand, crude extract was less cytotoxic than the phenolic extract by exhibiting higher IC_50_ values than the 5-FU. 5-FU was chosen as a reference drug due to its potent chemotherapeutic activity for the treatment of solid cancers.

We suggest that these findings could be attributed to the high content of certain phenolic compounds such as thonningianin-A (145 mg were obtained from 1.5 g of *T. sanguinea* subaerial extract) [27], gallic acid (20 mg were obtained from 20 g of *T. sanguinea* flower extract), brevifolin carboxylic acid (17 mg were obtained from 20 g of *T. sanguinea* flower extract) [26], and (+)-epipinoresinol (100 mg was obtained from 423 g extract of *T. sanguinea*) [28]. To confirm this point of view, it was previously reported that thonningianin-A has a potent antihepatocarcinoma activity in a dose-dependent manner. It is an effective antitumor constituent capable of inhibiting the proliferation of HepG-2 cells by apoptosis induction through caspase activation [33,34]. Additionally, gallic acid showed a potential cytotoxic activity against MCF-7 human breast cancer cells through the activation of caspases, which are responsible for programmed cell death, suggesting its probable use as antitumor agents [35,36]. Similarly, brevifolin carboxylic acid had potent cytotoxicity against human lung adenocarcinoma cells (IC_50_ = 3.95 µg/mL), which was almost similar to the IC_50_ value (95 µg/mL) against mouse leukemia cell [37]. Moreover, (+)-epipinoresinol showed significant inhibitory activity against human hepatoma (HepG2), human ovary serous adenocarcinoma (HO-8910), and human bladder cancer (T24) [38].

#### 2.2.3. Apoptosis Investigation

Phenolic extract exhibited promising cytotoxic activities against both MCF-7 and HepG2 cancer cell lines, so apoptosis investigation with flow cytometric and RT-PCR analyses in MCF-7 cells treated with phenolic extract (16.67 μg/mL, 48 h) were investigated. 

##### Annexin-V-FITC/PI Staining and Cell Cycle Analysis

**Figure 3:**
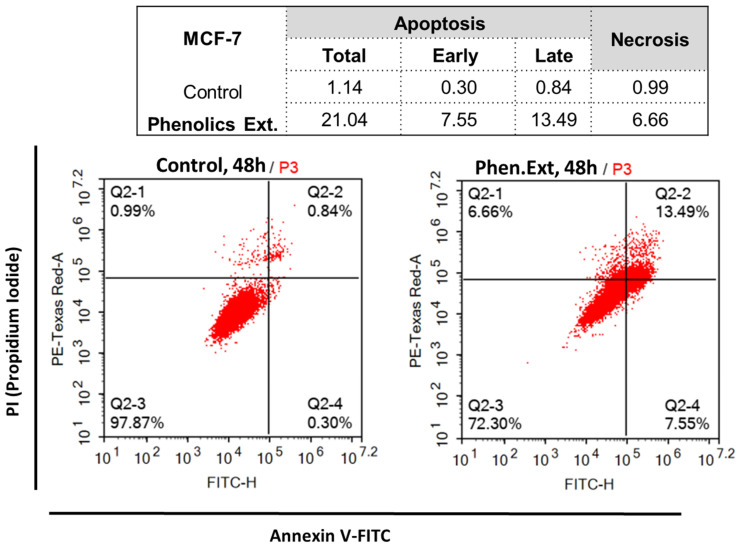
FITC/Annexin-V-FITC/PI differential apoptosis/necrosis of untreated and treated MCF-7 with phenolic extract at IC_50_ of 16.67 μg/mL, 48 h. Quadrant charts show Q-2-1 (necrosis, AV–/PI+), Q-2-2 (late apoptotic cells, AV+/PI+), Q-2-3 (normal cells, AV–/PI–), and Q-2-4 (early apoptotic cells, AV+/PI–).

Untreated and treated MCF-7 cells with phenolic extract were investigated for apoptotic activity. As shown in Figure 3, phenolic extract treatment induced total apoptosis in MCF-7 cells by 21.04 %, compared to 1.14 % in the control. It induced early apoptosis by 7.55 %, and late apoptosis by 13.49 %, while phenolic extract induced necrotic cell death by 6.66 %, compared to 0.99% in the control. These results illustrate apoptosis induction rather than necrosis as the mechanism of cytotoxic activity in MCF-7 cells.

**Figure 4:**
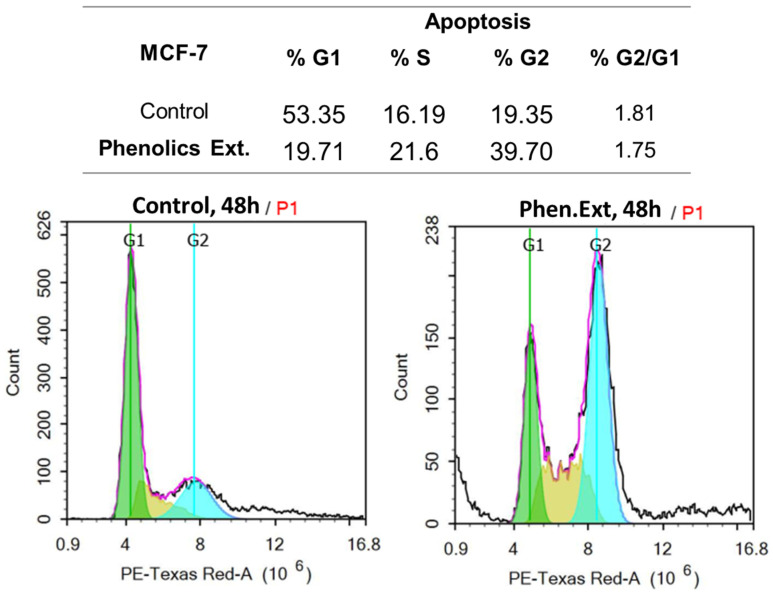
DNA content–flow-cytometry-aided cell cycle analyses of untreated and treated MCF-7 with the phenolic extract at IC_50_ of 16.67 μg/mL, 48 h.

In this study, MCF-7 cells untreated and treated with the phenolic extract were subjected to the DNA flow to determine the cell population in each cell phase and to determine at which cell cycle cells were arrested. As seen in Figure 4, phenolic extract treatment significantly increased the G2 population by 39.7 %, compared to 19.35 % for the control, while it significantly decreased the G1 population by 19.71%, compared to 53.35 %. Hence, phenolic extract treatment may induce apoptotic cell death in the G2/M phase. These data, regarding apoptotic investigation in MCF-7 cells upon treatment, agree with previous studies [39,40], which exhibited a higher ratio of apoptosis over necrosis and illustrated G2/M as the phase at which cell cycle was arrested.

##### RT-PCR

**Figure 5:**
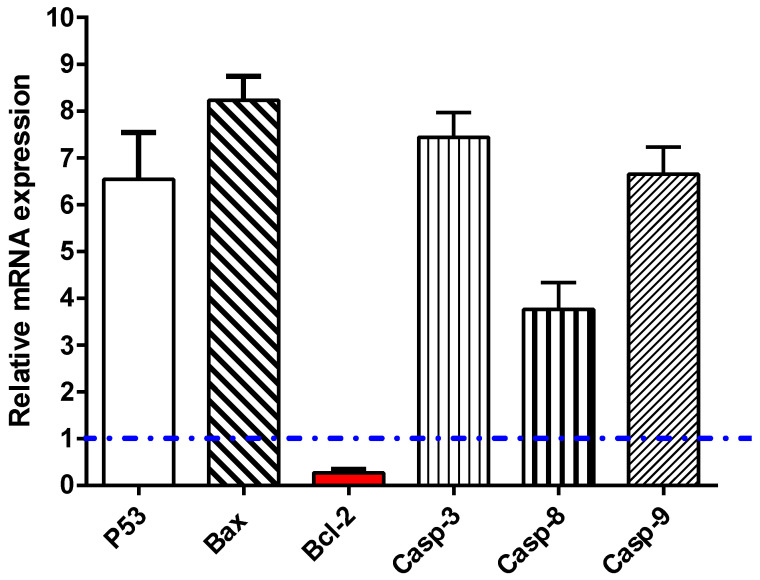
RT-PCR of apoptosis-related genes in MCF-7 untreated and treated with the phenolic extract at IC_50_ of 16.67 μg/mL, 48 h.

To investigate the apoptotic pathway of the phenolic extract, 16.67 µg/mL of the sample was added to MCF-7 cells and left for 48 h; after RNA extraction, cDNA was produced. Then, the mRNA expression of caspases 3, 8, and 9, and proapoptotic (P53 and Bax) as well as anti-apoptotic genes (Bcl-2) in the treated cells, was traced by RT-PCR analysis. As demonstrated in Figure 5, the expression of the P53 gene was noticeably elevated by the phenolics extract (≈6.54-fold), with concomitant activation of the Bax and caspases 3, 8, and 9, by 8.2-fold, 7.44-fold, 3.76-fold, and 6.65-fold, respectively. Meanwhile, it markedly suppressed the expression of Bcl-2 (the anti-apoptotic gene) by 0.27-fold. These findings are in harmony with the apoptotic mechanism suggested for anti-cancer activity.

In agreement with the apoptotic investigation of the flow cytometric results, the RT-PCR results exhibit upregulation of the proapoptotic proteins P53, Bax, and caspase-3, 8, and 9, and downregulation of anti-apoptotic proteins (Bcl-2) in the MCF-7 cells untreated and treated with phenolic extract. Caspases are key components in apoptosis in various cells and their activation is a vital episode in the progression of apoptosis, while the mitochondrial cytochrome c and caspase activation are controlled by Bcl-2 proteins.

### 2.3. LC-ESI-TOF-MS/MS Analysis

**Figure 6:**
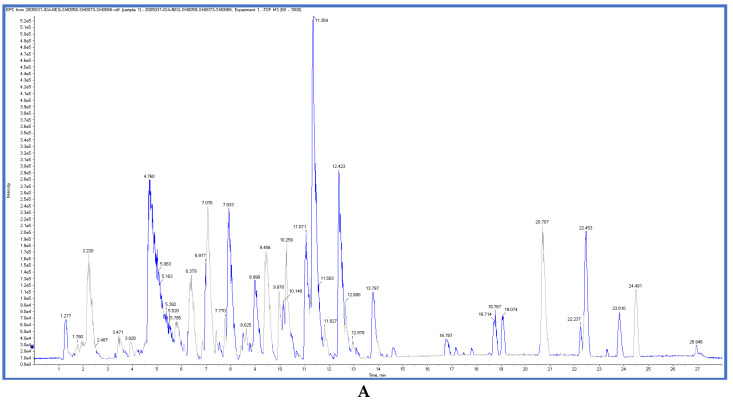

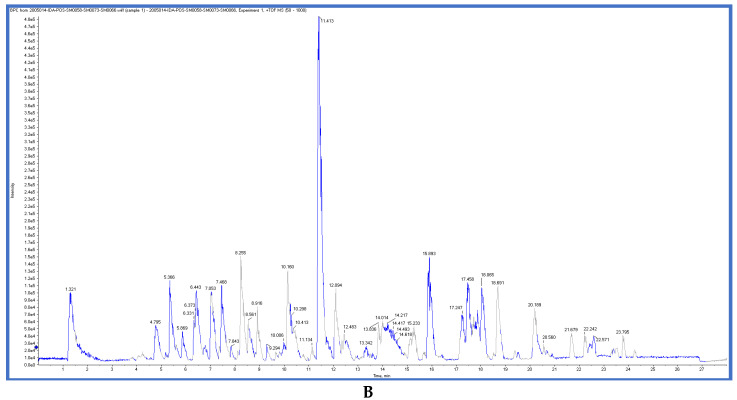
Chromatogram of methanolic extract of *T. sanguinea* in negative ion mode (**A**) and positive ion mode (**B**).

*T. sanguinea* extract was previously investigated for its chemical constituents. As a result, 25 compounds were isolated and identified [27,28]. The structures of the previously isolated compounds are illustrated in Supplementary Materials Figure S1. In the current study, LC-ESI-TOF-MS/MS analysis was applied to the crude methanolic extract of the plant to investigate the additional chemical constituents (Figure 6). As a result, 17 compounds were revealed, including ten compounds detected in the negative ion mode (Table 2) and seven compounds detected in the positive ion mode (Table 3).

### 2.4. Molecular Docking Studies

In this present study, *T. sanguinea* phenolic extract exhibited antiproliferative activity against breast cancer cells via apoptosis. Hence, all compounds, whether identified by the present LC-ESI-TOF-MS/MS analysis or previously isolated from the plant, were chosen for a simulated molecular docking investigation to gain insights into the possible molecular targets for the cytotoxic and apoptosis-inducing activities. As seen in Table 4 and Table S3, most of the identified compounds revealed good binding interactions with the key amino acids inside the “B-cell lymphoma 2 (Bcl-2), PDB ID: 4IEH”, while there were some minor compounds that did not show any binding activity towards the studied target. Interestingly, compounds thonningianin-A and -B were promising compounds, possessing strong interactions with the key amino acids (mainly Arg 66) of the binding site, as shown in Table 4 with 2D and 3D disposition images. Accordingly, our docking experiment proposed their mechanism of action as Bcl-2 suppressors, congruent with the flow cytometric and RT-PCR analyses, illustrating their apoptosis-inducing activity. It is worth mentioning that thonningianin-A is a major active constituent of *T. sanguinea*, as it was isolated in an amount of 145 mg from 1.5 g of the plant extract [27]. Our rationalization for targeting Bcl-2 as the molecular target for the phenolic extract agreed with the study of Verma et al. [57], which developed some natural polyphenolics as inhibitors against the anti-apoptotic Bcl-2 gene.

## 3. Materials and Methods

### 3.1. Plant Material

*T. sanguinea* whole plant was collected from the eastern region of Ghana in January 2015. The plant was authenticated by the curator of the herbarium. A voucher specimen was deposited, labeled with the code CSRPM no. 140.

### 3.2. Preparation of Plant Extract

The whole plant of *T. sanguinea* (300 g) was shade dried for seven days and pulverized. The dried powdered plant material was extracted by cold maceration with MeOH (3 × 1 L for 3 days), followed by MeOH:CHCl_3_ (1:1; 3 × 1 L for 3 days) at room temperature. The filtrates were pooled together and concentrated in vacuo using the rotary evaporator (BÜCHI Labortechnik AG, Flawil, Switzerland) to produce 20 g of dry residue.

### 3.3. Determination of Total Phenolic Content

The Folin–Ciocalteu method [58] was used to determine total phenolic content. Briefly, sample solution (0.5 mL) was mixed with 2.5 mL of Folin–Ciocalteu reagent, and 2 mL of sodium carbonate was then added. After incubation at room temperature for 2 h, the absorbance of the reaction mixture was measured at 760 nm against a blank using a Spectrophotometer UV/Vis specor D 250 plus (Analytik Jena GmbH, Jena, Germany). Gallic acid was used as the standard, and the total phenolic content was expressed as mg/g, calculated as gallic acid equivalents

### 3.4. Preparation of Phenolic Extract

*T. sanguinea* phenolic compounds were extracted using 200 g of the powdered plant by treatment with 5% Na_2_CO_3_ aqueous solution, which was left for one hour. Then, the mixture was filtered and washed with distilled water. The filtrate was diluted with distilled water, neutralized using HCl, then partitioned between chloroform, ethyl acetate, and n-butanol. The obtained extracts were combined, then concentrated under reduced pressure using a rotary evaporator (BÜCHI Labortechnik AG, Flawil, Switzerland) [59].

### 3.5. Determination of Antioxidant Activity

#### 3.5.1. Initial Screening Step

Solutions of the provided samples were prepared in final concentrations of 1000 and 100 µg/mL in MeOH, to identify the range within which the inhibitory concentration 50 (IC_50_) lies.

#### 3.5.2. Determination of IC_50_

Extracts that exceeded 50% inhibition in any of the initial screening step concentrations were serially diluted to provide five concentrations. 

#### 3.5.3. Trolox Standard Preparation

A stock solution of 100 µM concentration of Trolox was prepared in methanol, from which 7 concentrations were prepared, including 50, 40, 30, 20, 15, 10, and 5 µM.

#### 3.5.4. DPPH^•^ Assay

A DPPH^•^ (2,2-diphenyl-1-picryl-hydrazyl-hydrate) free radical assay was carried out according to the previously described method [60]. Briefly, 100 µL of freshly prepared DPPH reagent (0.1% in methanol) was added to 100 µL of the sample in a 96-well plate (*n* = 6), and the reaction was incubated at room temperature for 30 min in dark. At the end of incubation time, the resulting reduction in DPPH^•^ color intensity was measured at 540 nm. The scavenging of the DPPH^•^ radical (PI) was determined, and the data are represented as means ± SD according to the following equation:

PI = [{(average absorbance of blank—average absorbance of the test)/average absorbance of blank} × 100]

The results were recorded using the microplate reader FluoStar Omega.

#### 3.5.5. Data Analysis

The data were analyzed using Microsoft Excel^®^, and the IC_50_ value was calculated using GraphPad Prism 5^®^ by converting the concentrations to their logarithmic value and selecting the nonlinear inhibitor regression equation (log (inhibitor) vs. normalized response—variable slope equation) [61].

### 3.6. LC-ESI-TOF-MS/MS Analysis

The mobile phase was prepared from DI water:methanol:acetonitrile in a ratio of (2:1:1), then 50 mg of dry methanolic extract was mixed with 1 mL of the mobile phase. The mixture was ultra-sonicated for 10 min and centrifuged for 10 min at 11,200× *g*. Finally, the injected concentration was adjusted to be 1 µg/µL. The injected volume was 25 µL in both positive and negative mode; in addition, 25 µL of the mobile phase was injected as a blank sample. MasterView was used for peaks extraction based on the following criteria: features should have a signal-to-noise ratio greater than 5, and the feature intensities of the sample-to-blank ratio should be greater than 5. A cutoff score of 70% was used to finely select the identified metabolites [23]. LC-ESI-TOF-MS/MS parameters and conditions are listed in the Supplementary Materials, Table S2.

### 3.7. Biological Evaluation

#### 3.7.1. Cytotoxic Activity

Cytotoxic activities of crude extract and phenolic extract were screened against breast cancers of MCF-7 (ATCC^®^ HTB-22™ Homo sapiens) and MDA-MB-231 (ATCC^®^ HTB-26™ Homo sapiens); liver HepG2 (ATCC^®^ HB-8065™ Homo sapiens) and lung A549 (ATCC^®^ CCL-185™ Homo sapiens) cancer cell lines. Each cell line was cultured in a proper complete medium, according to the standard cell culture work. Following routine MTT assay, the treatment of cells with crude extract and phenolic extract for 48 h incubation was carried out, and standard fluorouracil (5-FU) was used. Cell viability percentages versus the log values of serial concentrations were calculated. Then, IC_50_ values in µg/mL were calculated using GraphPad Prism 7 [62].

#### 3.7.2. Apoptotic Investigation 

##### Flow Cytometric Analysis

Flow cytometric analyses: “FITC/Annexin-V-FITC/PI, and DNA content cell cycle” were performed following previously described methods. The detailed methodology is provided in the Supplementary Materials. In brief, MCF-7 cells were treated with phenolic extract (16.54 μg/mL, 48 h), and 10^5^ cells were harvested by trypsinization and washed two times with ice-cold PBS (pH = 7.4). This was followed by the addition of 500 μL Annexin V-FITC/PI solution to the cells. Then, cells were injected into the flow cytometer. Finally, ACEA NovoExpress™ software (ACEA Biosciences Inc., San Diego, CA, USA) was employed for quadrant analysis of the produced data, to estimate the percentage of apoptotic cells, and to determine at which phase cell cycle would be arrested [39].

##### RT-PCR 

Phenolic extract (16.54 μg/mL, 48 h) was treated for the MCF-7 cells. Cells were collected at the end of the treatments, and total RNA was extracted with the RNeasy^®^ Mini Kit (cat.no. 74104, Hilden, Germany), as directed by the factory manufacturer. The cDNA synthetic kit (Bio-Rad, Hercules, CA, USA) was performed with 500 ng of RNA by i-Script cDNA synthesis, as instructed by the manufacturer. All reactions were performed for 35 cycles using this temperature profile: 95 °C for 5 m (initial denaturation); 95 °C for 15 min (denaturation), 55 °C for 30 min (annealing), and 72 °C for 30 min (extension). The cycle threshold values (Ct) and the relative folds of change between all samples were collected [40]. The primer sequences are listed in Table S2. 

#### 3.7.3. Molecular Docking

The molecular docking study was performed on the identified compounds (phenolic extract) towards the apoptosis inhibitor, Bcl-2 (PDB: 4IEH), to elucidate their cytotoxic and apoptotic activity profiles. The molecular docking studies were carried out using the MOE 2008–10 (Chemical Computing Group, Canada) as the computational software. The protein structure complexed with the co-crystallized ligand was easily accessible from the Protein Data Bank. Routine work concerning receptor and ligand preparation and molecular docking was carried out as previously published [63,64].

## 4. Conclusions

Herein, the chemical profile and cytotoxic activity of *Thonningia sanguinea* were mechanistically investigated. Using LC-ESI-TOF-MS/MS analysis, 17 hits were newly identified in the plant extract. We conclude that there was promising cytotoxic activity of the phenolic extract of *T. sanguinea* in MCF-7 and HepG2 cell lines, with IC_50_ 16.54 and 13.54 μg/mL, and it exhibited promising apoptotic activity in MCF-7 cells, with an 18.45-fold increase compared to the untreated control, arresting the cell cycle at G2/M. Furthermore, the molecular docking study revealed the promising binding activity of the chemical constituents of the Bcl-2 inhibitor, and this agrees with the RT-PCR results, which show the upregulation of the proapoptotic genes and downregulation of the anti-apoptotic genes. Hence, future fractionation studies are recommended for the phenolic extract, in order to specify the most active compound, and it should be tested using an in vivo animal model for the development of a novel anti-cancer agent from this plant.

## Figures and Tables

**Table 1:** IC_50_ (μg/mL) * values of both crude and phenolic extracts against panels of breast, liver, and lung cancer using the MTT assay.

Tested Samples	IC_50_ (μg/mL) *			
	A549	HepG2	MDA-MB-231	MCF-7
Crude extract	13.25 ± 0.46	18.76 ± 0.98	56.53 ± 1.32	34.65 ± 1.76
Phenolic extract	≥50	13.51 ± 0.54	≥50	16.67 ± 0.87
5-FU	7.47 ± 0.35	15.8 ± 0.86	20.76 ± 0.76	26.98 ± 0.43

* Values are expressed as mean ± SD of 3 independent trials (*n* = 3). IC_50_ were calculated using GraphPad Prism 7 software using nonlinear regression dose–inhibition curve fit.

**Table 2:** Chemical constituents of *T. sanguinea* extract newly identified by LC-ESI-TOF-MS/MS analysis (negative ion mode).

Compound	RT (min)	Measured *m/z* [M−H]^−^	Expected *m/z* [M−H]^−^	Relative Error *	Molecular Formula	Fragments
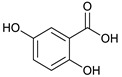 gentisic acid [41]	1.31	153.0155	153.0188	−21.56	C_7_H_6_O_4_	153, 108
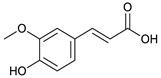 ferulic acid [42]	2.06	193.0528	193.0501	13.98	C_10_H_10_O_4_	178, 134
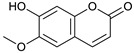 Scopoletin [42]	5.01	191.0332	191.0344	−6.28	C_10_H_8_O_4_	176, 147
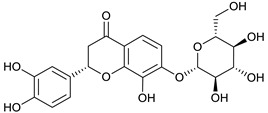 Isookanin-7-glucoside [43]	5.23	449.1062	449.1084	−4.89	C_21_H_22_O_11_	287, 151, 135,
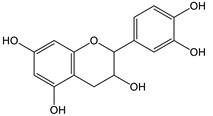 (+)-epicatechin [44]	7.06	289.0716	289.0712	1.38	C_15_H_14_O_6_	125, 123
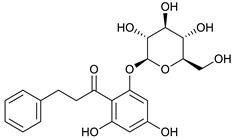 4-deoxyphloridzin [45]	7.85	419.1382	419.1342	9.54	C_21_H_24_O_9_	419, 257
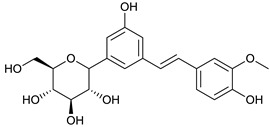 (isorhapontin) [44]	9.95	419.1328	419.1342	−3.34	C_21_H_24_O_9_	281,257
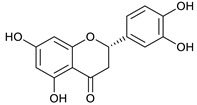 (+)-eriodictyol [46]	9.97	287.0565	287.0556	3.13	C_15_H_12_O_6_	151, 135
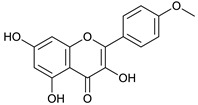 3, 5, 7-trihydroxy-4’-methoxyflavone [47]	11.74	299.0541	299.0556	−5.01	C_16_H_12_O_6_	299, 284, 256
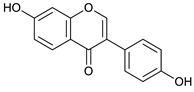 Daidzein [48]	18.78	253.0524	253.0501	9.08	C_15_H_10_O_4_	225, 224

* Mass accuracy was calculated as [measured mass-expected mass/expected mass] × 10^6^ and is expressed in parts per million (ppm) error [49].

**Table 3:** Chemical constituents of *T. sanguinea* extract newly identified by LC-ESI-TOF-MS/MS analysis (positive ion mode).

Compound	RT (min)	Measured *m/z* [M−H]^−^	Expected *m/z* [M−H]^−^	Relative Error *	Molecular Formula	Fragments
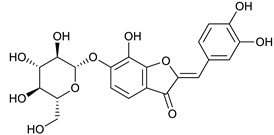 Maritimetin-6-*O*-glucoside [50]	6.50	449.1066	449.1084	−4.00	C_21_H_20_O_11_	449, 287
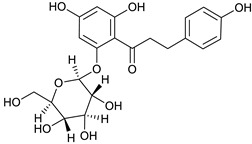 Phlorizin [51]	6.78	437.1446	437.1448	−0.45	C_21_H_24_O_10_	437, 275
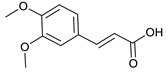 3,4-dimethoxycinnamic acid [52]	6.91	209.081	209.0814	−1.91	C_11_H_12_O_4_	209, 191, 163
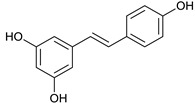 Resveratrol [53]	7.39	229.0853	229.0865	−5.23	C_14_H_12_O_3_	229, 185, 157
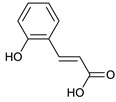 *trans ortho* Coumaric acid [54]	7.45	165.054	165.0552	−7.27	C_9_H_8_O_3_	165, 147, 91
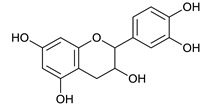 (-)-Epicatechin [55]	7.46	291.0873	291.0869	1.37	C_15_H_14_O_6_	165, 123
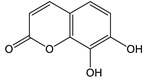 Daphnetin [56]	8.08	179.0342	179.0344	−1.11	C_9_H_6_O_4_	179, 133

* Mass accuracy was calculated as [measured mass-expected mass/expected mass] × 10^6^ and is expressed in parts per million (ppm) error [49].

**Table 4:** Summary of the ligand–receptor interactions of the promising docked compounds inside the active site of Bcl-2 (PDB: 4IEH).

Compound	Ligand-Receptor Interactions (HB and Van Der Waals Interactions)			
Thonningianin-A	Arg 66 Ala 59 Gly 104 Tyr 161 Asn 102		1 HB-Acceptor 1 HB-donor 1 HB-Acceptor 1 HB-Acceptor 1 HB-Acceptor	
	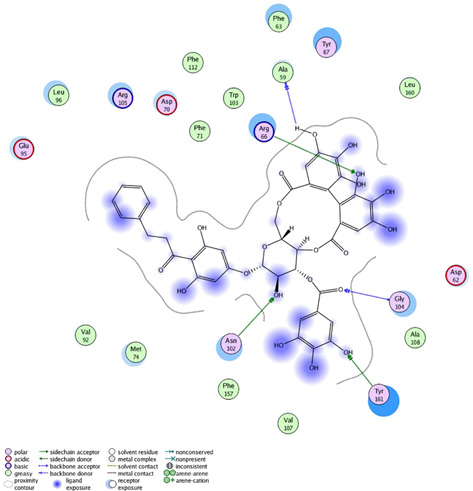			
	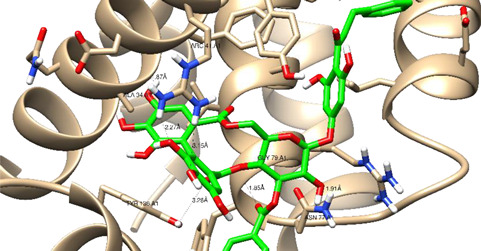			
Thonningianin-B	Arg 105 Gly 104 Asp 70 Glu 95	1 HB-Acceptor 1 HB-Donor 1 HB-Donor 1 HB-Donor		1 arene-cation with Arg 66 1 arene-arene with Tyr 161
	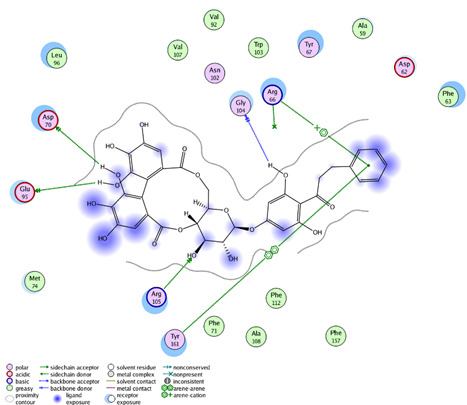			
	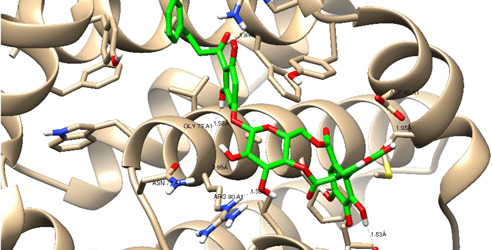			

* Analysis of the ligand–receptor interactions of the rest of the identified compounds are provided in the Supplementary Materials.

## Supplementary Materials

The following are available online at https://www.mdpi.com/article/10.3390/plants10102156/s1, Table S1. LC-ESI-TOF-MS/MS parameters and conditions; Table S2. Forward and reverse primers used in gene expression analysis; Table S3. Full report of the ligand–receptor interactions of the identified compounds inside the active site of Bcl-2 (PDB: 4IEH); Figure S1. Chemical structures of the compounds previously isolated from *T. sanguinea*.
