# Supplementary material for: Thonningia sanguinea Extract: Antioxidant and Cytotoxic Activities Supported by Chemical Composition and Molecular Docking Simulations
Source: Plants (Basel). 2021 Oct 11;10(10):2156. doi: 10.3390/plants10102156 (PMC8539418; doi:10.3390/plants10102156)
Supplement: Supplementary file 1 [file plants-10-02156-s001.zip › plants-1393230-supplementary.pdf]

Thonningia sanguinea Extract: Antioxidant and Cytotoxic Activities Supported by Chemical Composition and Molecular Docking Simulations

Supplementary materials for detailed methodology

Table S1. LC-ESI-TOF-MS/MS parameters and conditions

**Mobile phase components:**	**Mode**	**Type**	-
Mobile phase A	Positive	5 mM ammonium formate buffer pH 3containing 1% methanol	
Mobile phase B	Negative	5 mM ammonium formate buffer pH8 containing 1% methanol	
Mobile phase C	Positive / Negative	100 % acetonitrile	
**Flow rate**	0.3 ml/min		
**Pump time table**			
**Time (min)**	**Flow ml/min**	**Mobile phase A (or B)**	**Mobile phase C**
0	0.3	90	10
1	0.3	90	10
21	0.3	10	90
25	0.3	10	90
25.01	0.3	90	10
28	0.3	90	10
**LC Column**	**Type**	**Vendor**	**Specs**
Pre column	In-Line filter disks	Phenomenex	0.5 µm x 3.0 mm
Column	X select HSS T3	Waters	2.5 µm, 2.1x150 mm
**Column temperature**	40 °C		
**Injection volume**	10 µl		
**Item**	**Type**	**Vendor**	**Utility**
ExionLC (High flow LC)	Hardware	Sciex	Chromatographic separation
TripleTOF 5600+	Hardware	Sciex	IDA acquisation
Analyst TF 1.7.1	Software	Sciex	LC-QTOF control
Reifycs Abf (Analysis Base File) Converter	Software	Reifycs	Wiff file conversion
MS-DIAL	Software	RIKEN	Data analysis

Flow cytometric analysis

FITC/Annexin-V-FITC/PI Differential Apoptosis/Necrosis Assessment

Apoptosis and necrosis cell populations are determined using Annexin V-FITC apoptosis detection kit (Abcam Inc., Cambridge Science Park, Cambridge, UK) coupled with 2 fluorescent channels flowcytometry. After treatment with test compounds for 48 h, cells (10^5 cells) are collected by trypsinization and washed twice with ice-cold PBS (pH 7.4). Then, cells are incubated in dark with 0.5 ml of Annexin V-FITC/PI solution for 30 min in dark at room temperature according to manufacturer protocol. After staining, cells are injected via ACEA Novocyte™ flowcytometer (ACEA Biosciences Inc., San Diego, CA, USA) and analyzed for FITC and PI fluorescent signals using FL1 and FL2 signal detector, respectively (λex/em 488/530 nm for FITC and λex/em 535/617 nm for PI). For each sample, 12,000 events are acquired and positive FITC and/or PI cells are quantified by quadrant analysis and calculated using ACEA NovoExpress™ software (ACEA Biosciences Inc., San Diego, CA, USA).

DNA Content-Flow Cytometry Aided Cell Cycle Analysis

After treatment with test compounds for 48 h, cells (10^5 cells) are collected by trypsinization and washed twice with ice-cold PBS (pH 7.4). Cells are re-suspended in two milliliters of 60% ice-cold ethanol and incubated at 4ºC for 1 h for fixation. Fixed cells are washed twice again with PBS (pH 7.4) and re-suspended in 1 mL of PBS containing 50 µg/mL RNAase A and 10 µg/mL propidium iodide (PI). After 20 min of incubation in dark at 37 C, cells are analyzed for DNA contents using flow cytometry analysis using FL2 (λex/em 535/617 nm) signal detector (ACEA Novocyte™ flowcytometer, ACEA Biosciences Inc., San Diego, CA, USA). For each sample, 12,000 events are acquired. Cell cycle distribution is calculated using ACEA NovoExpress™ software (ACEA Biosciences Inc., San Diego, CA, USA).

Gene expression using RT-PCR

MCF-7 cells were treated at 37 °C with the IC_{50} value of the phenolic extract (16.54 µg/ml, 48h) and were collected for real-time PCR analysis with the perfect primer pairs (Supplementary, Table 1) of the following eight genes (P53, PUMA, BCL2, CASP3, CASP8, CASP9, PI3k and AKT) compared to the control cells, and for analysis first total RNA was extracted using RNeasy Mini Kit (Qiagen) following the manufacturer protocol of the kits than RNA was assessed for both the quality and quantity of RNA using NanoDrop ND-1000 spectrophotometer at the absorbance ratio 260/280 nm [NanoDrop Tech., Inc. DE, USA]. Also, Whole RNA quality was examined by 1% agarose gel electrophoresis and afterward cDNA was obtained from one µg RNA using I-Script cDNA synthesis kit (BioRad). qRT-PCR were executed utilizing 10 ng of cDNA and Applied Biosystems Sybr Green master mix [USA]. Primers utilized during real time PCR was listed in table (1) and the cycling conditions were 5 min [95

°C], followed by 45 cycles for 15 s [95 °C], for 60 s [58, 60, 62 °C] and for 1 min [72 °C] using applied Biosystems Step-One qRT-PCR (USA). Relative genes expression was finally calculated utilizing the ΔΔCT equation with normalization to that of the housekeeping beta-actin gene. Results were shown as fold change in gene expression in the treated cells versus that of the control

Table S2. Forward and reverse primers used in gene expression analysis.

Gene	Forward	Reverse
**P53**	5'- CCCCTCCTGGCCCCTGTCATCTTC-3'	5'- GCAGCGCCTCACAACCTCCGTCAT -3'
**PAX**	5′-GTTTCATCCAGGATCGAGCAG-3′	5′-CATCTTCTTCCAGATGGTGA-3′
**CASP-3**	5'- TGGCCCTGAAATACGAAGTC-3'	5'- GGCAGTAGTCGACTCTGAAG -3'
**CAS-8**	5'- AATGTTGGAGGAAAGCAAT -3'	5'- CATAGTCGTTGATTATCTTCAGC -3'
**CASP-9**	5'- CGAACTAACAGGCAAGCAGC -3'	5'- ACCTCACCAAATCCTCCAGAAC -3'
**BCL2**	5′- CCTGTGGATGACTGAGTACC -3′	5′- GAGACAGCCAGGAGAAATCA -3′
**β-actin**	5′- GTGACATCCACACCCAGAGG -3`	5′- ACAGGATGTCAAAACTGCCC -3′

Molecular docking

Table S3. Full report of ligand-receptor interactions of the identified compounds inside the active site of BCL2 (PDB: 4IEH)

Compound	Structure	Ligand-receptor interactions inside the active site of BCL2 (PDB: 4IEH)
**2,5-Dihydroxybenzoic acid**	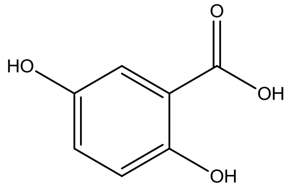	---
**3-(4-hydroxy-3-methoxyphenyl) prop-2-enoic acid**	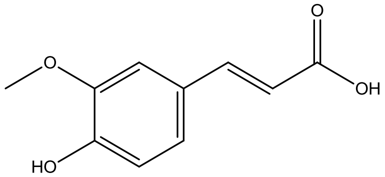	**Arg 105**<br>**Glu 95**<br>1 arene-arene with Tyr 161
**Scopoletin**	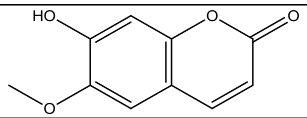	---

**Isookanin-7-glucoside**	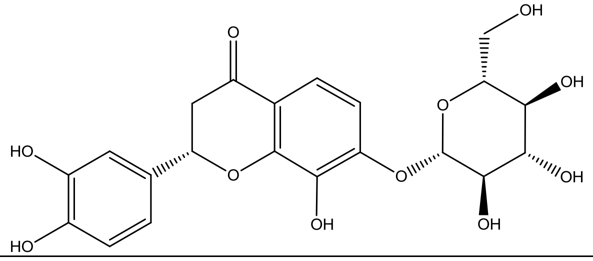	**Gly 104**<br>**Asp 70**<br>1 arene-arene with Tyr 161
**(+)-3 3' 4' 5 7-Pentahydroxyflavan**	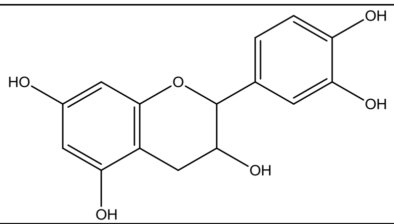	**Gly 104**<br>**Glu 95**<br>1 arene-cation with Arg 66
**4-deoxyphloridzin**	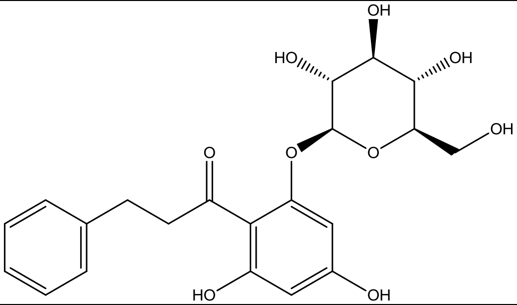	**Gly 104**<br>**Glu 95**<br>1 arene-cation with Arg 66
**E-4,5'-dihydroxy-3-methoxy-3'-glucopyranosylstilbene**	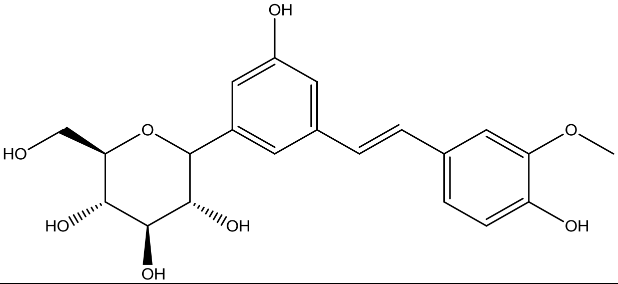	**Arg 105**<br>**Asp 70**<br>1 arene-arene with Tyr 161
**3, 5, 7-trihydroxy-4'-methoxyflavone**	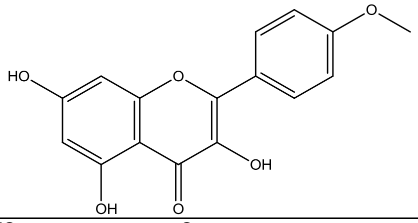	**Arg 105**<br>**Glu 95**<br>1 arene-cation with Arg 66
**Daidzein**	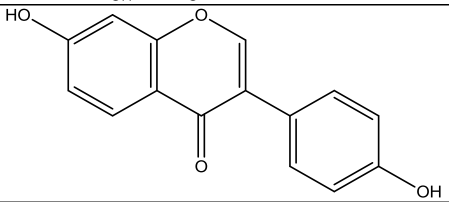	**Arg 105**<br>**Glu 95**

**Maritimetin-6-*O*-glucoside**	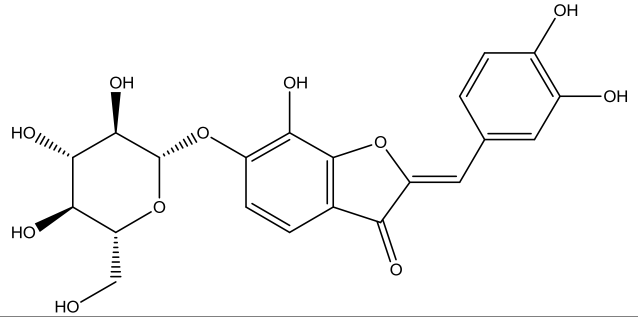	**Arg 105**<br>**Gly 104**<br>1 arene-arene with Tyr 161
**Phlorizin**	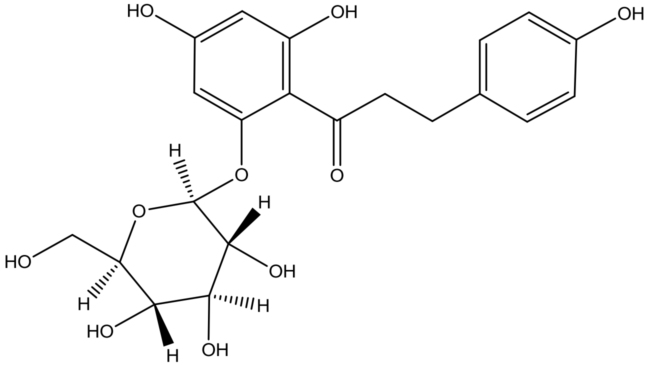	**Arg 105**<br>**Glu 95**<br>1 arene-cation with Arg 66
**3,4-dimethoxycinnamic acid**	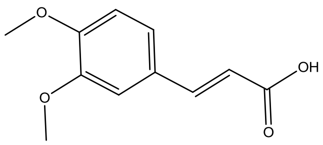	--
**Resveratrol**	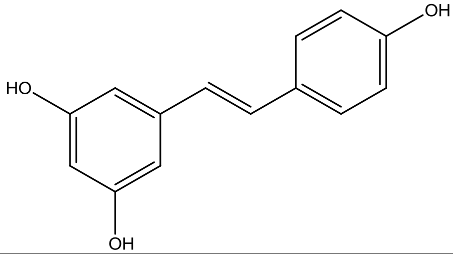	**Arg 105**<br>1 arene-arene with Tyr 161
**Trans ortho coumaric acid**	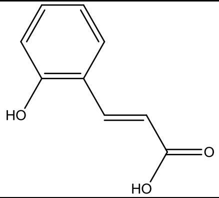	--
**(-)-Epicatechin**	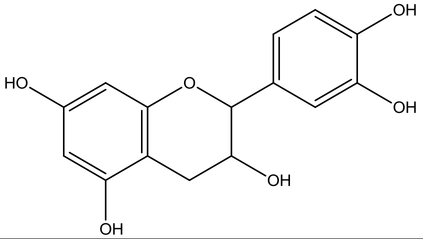	**Asp 70**<br>1 arene-arene with Tyr 161
**Daphnetin**	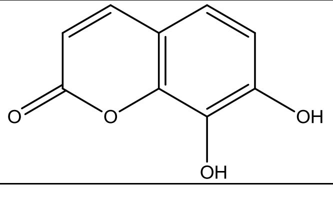	---

**3-hydroxyphloridzin**	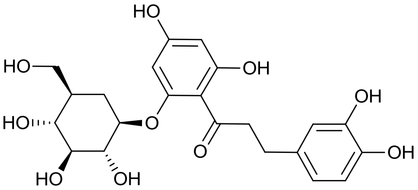	**Arg 105**<br>**Gly 104**<br>1 arene-arene with Tyr 161
**2', 6'-dihydroxy-4'-(*β*-D-glucopyranosyloxy) dihydrochalcone**	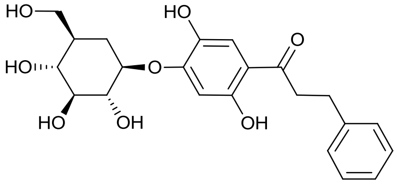	**Arg 105**<br>**Gly 104**<br>1 arene-cation with Arg 66
**2'-*O*-(6-*O*-galloyl- *β* -D-glucopyranosyl)-3-hydroxyphloretin**	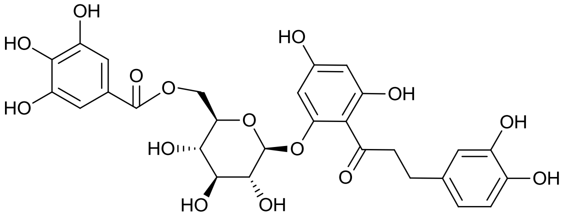	**Arg 105**<br>**Gly 104**<br>1 arene-cation with Arg 66
**2'-*O*- (3-*O*-galloyl-4,6-*O*-Sa-hexahydroxydiphenoyl- *β* -D-glucopyranosyl)-3-hydroxyphloretin.**	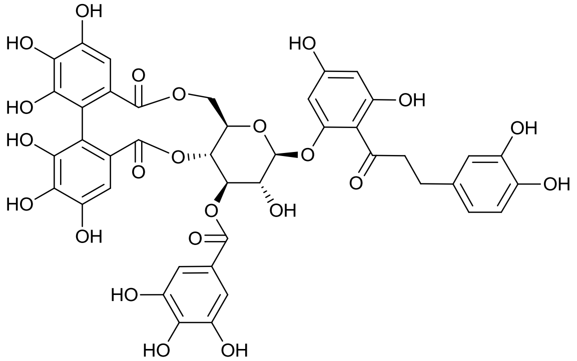	**Arg 105**<br>**Gly 104**<br>1 arene-cation with Arg 66
**2',6'-dihydroxy-4'-*O*-(6-*O*-galloyl- *β* -D-glucopyranosyl) dihydrochalcone**	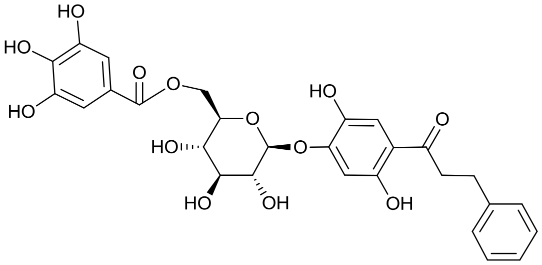	**Arg 105**<br>**Gly 104**<br>1 arene-cation with Arg 66
**2'-*O*-(4,6-*O*-Sa-hexahydroxydiphenoyl- *β* -D-glucopyranosyl)-3-hydroxyphloretin**	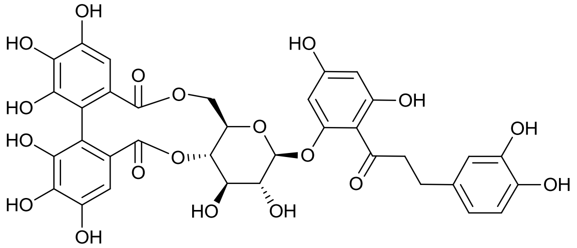	**Arg 105**<br>**Gly 104**<br>1 arene-cation with Arg 66

**2'-*O*-(3-*O*-galloyl-4,6-*O*-Sa-hexahydroxydiphenoyl- *β* -D-glucopyranosyl)phloretin.**	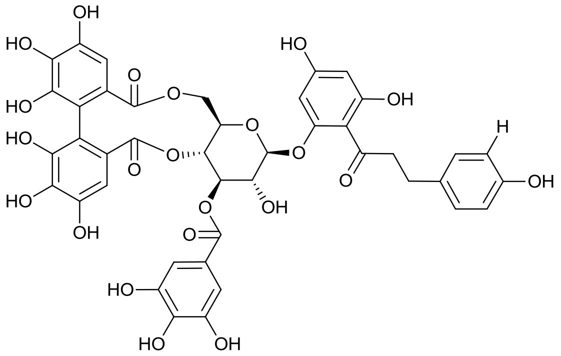	**Arg 105**<br>**Gly 104**<br>1 arene-arene with Tyr 161
**4'-*O*-(4,6-*O*-Sa-hexahydroxydiphenoyl- *β* -D-glucopyranosyl)- phloretin**	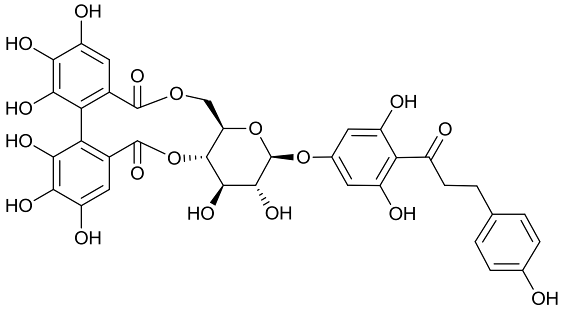	**Arg 105**<br>**Glu 95**<br>1 arene-arene with Tyr 161
**Brevifolincarboxylic acid**	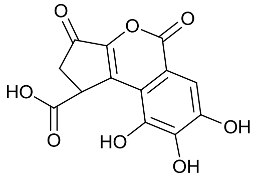	1 arene-arene with Tyr 161
**gallic acid**	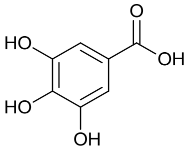	--
**(+)-Epipinoresinol**	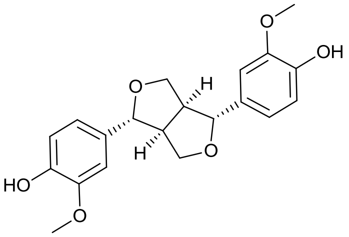	1 arene-cation with Arg 66<br>1 arene-arene with Tyr 161
**(+)-Eriodictyol**	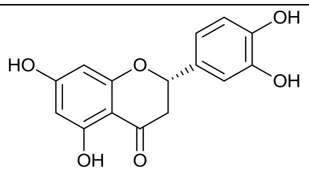	1 arene-cation with Arg 66<br>1 arene-arene with Tyr 161
**(+)-pinoresinol**	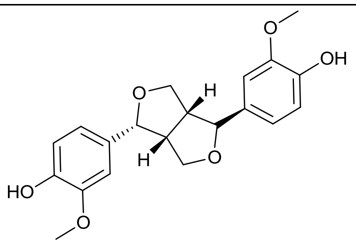	**Arg 105**<br>**Asp 70**<br>1 arene-arene with Tyr 161

**cycloolivil**	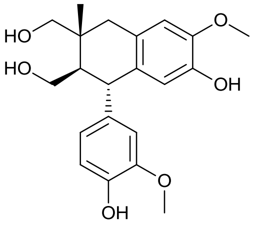	**Arg 105**<br>**Glu 95**<br>1 arene-arene with Tyr 161
**(+)-secolariciresinol**	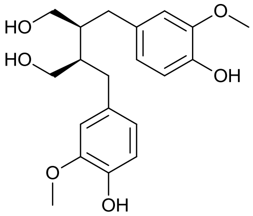	**Arg 105**<br>**Asp 70**
**(+)-Isolariciresinol**	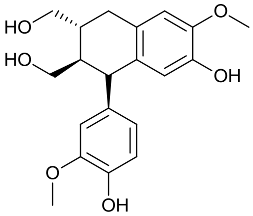	**Asp 70**<br>**Glu 95**

Figure S1. Chemical structures of the compounds previously isolated from *T. sanguinea*

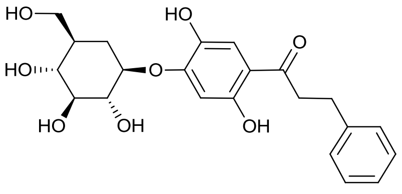

2', 6'-dihydroxy-4'-(β-D-glucopyranosyloxy) dihydrochalcone

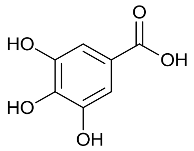

gallic acid

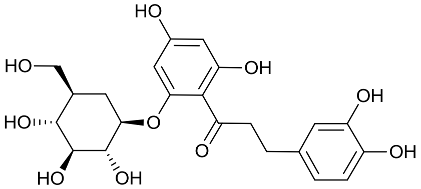

3-hydroxyphloridzin

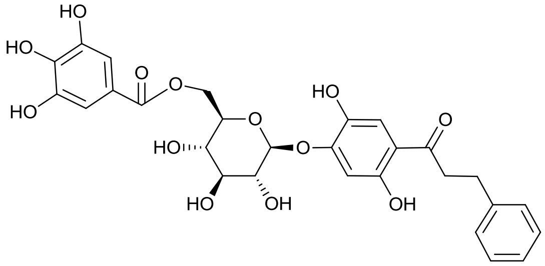

2',6'-dihydroxy-4'-*O*-(6-*O*-galloyl- β -D-glucopyranosyl) dihydrochalcone

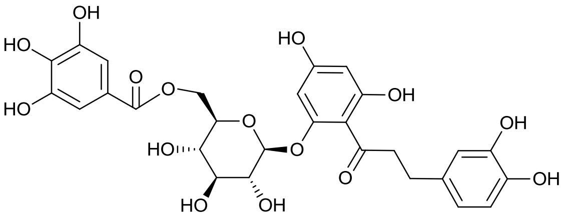

2'-*O*-(6-*O*-galloyl- β -D-glucopyranosyl)-3-hydroxyphloretin

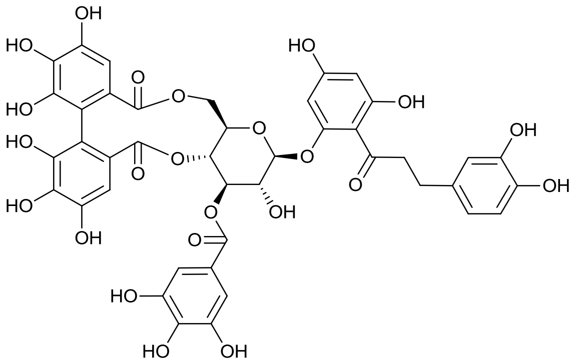

2'-*O*- (3-*O*-galloyl-4,6-*O*-Sa-hexahydroxydiphenoyl- β -D-glucopyranosyl)-3-hydroxyphloretin.

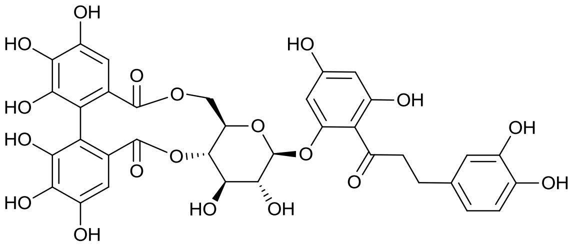

2'-*O*-(4,6-*O*-Sa-hexahydroxydiphenoyl- β -D-glucopyranosyl)-3-hydroxyphloretin

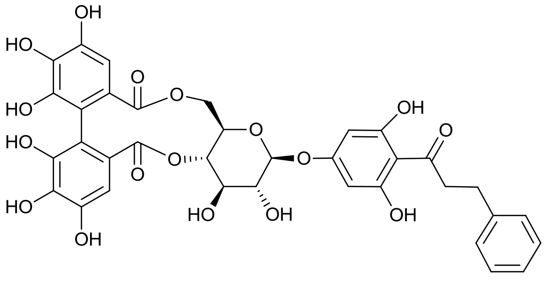

thonningianin B

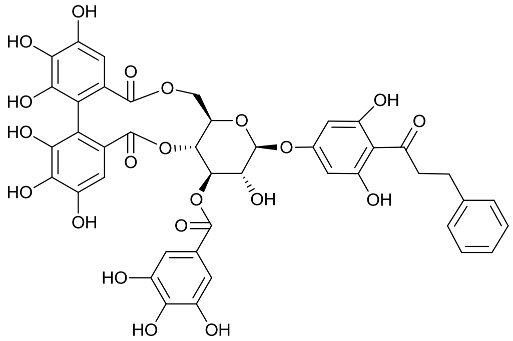

thonningianin A

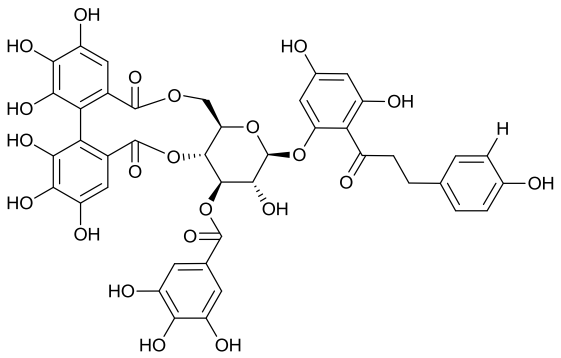

2'-*O*-(3-*O*-galloyl-4,6-*O*-Sa-hexahydroxydiphenoyl- *β* -D-glucopyranosyl)phloretin.

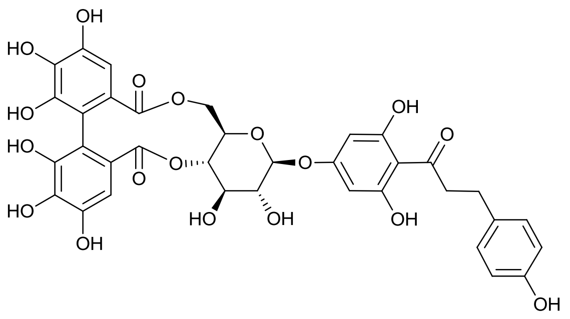

4'-*O*-(4,6-*O*-Sa-hexahydroxydiphenoyl- *β* -D-glucopyranosyl)- phloretin

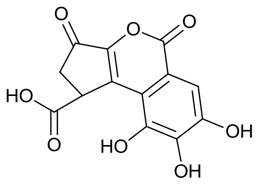

brevifolincarboxylic acid

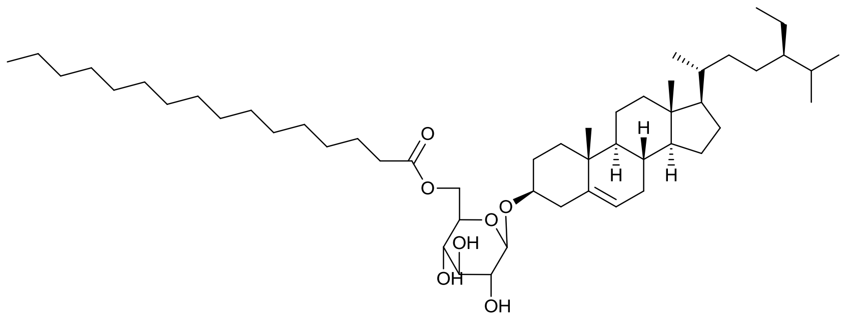

***β* -sitosteryl-3 *β* -D-glucopyranoside-6'-*O*-palmitate**

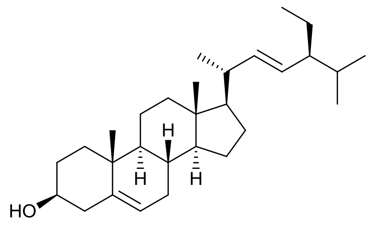

stigmasterol

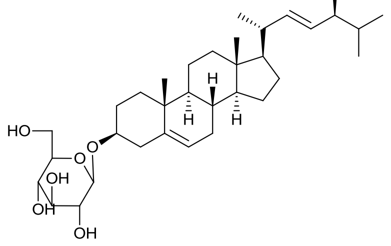

stigmasterol glucoside

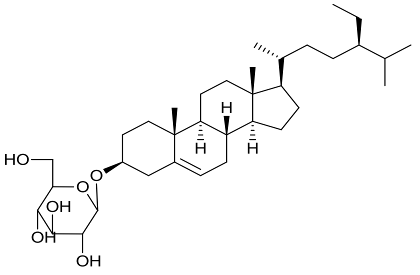

***β* -sitosterol glucoside**

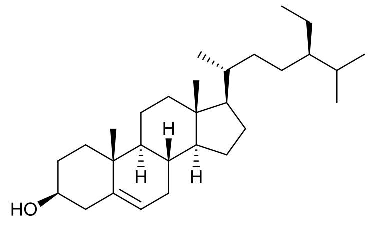

***β* -sitosterol**

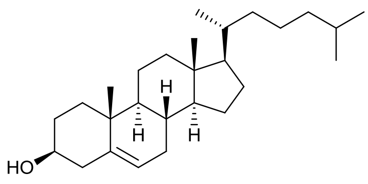

cholesterol

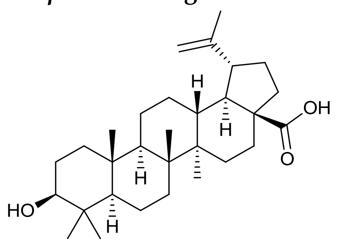

betulinic acid

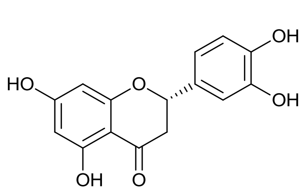

(+)-eriodictyol

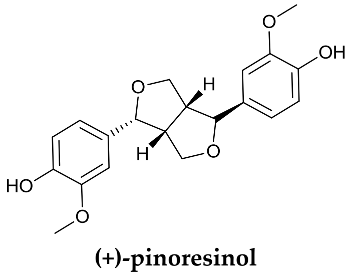

(+)-pinoresinol

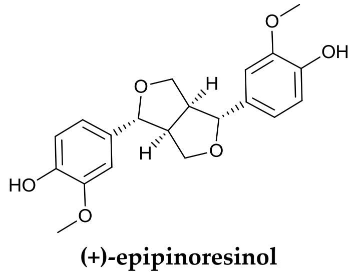

(+)-epipinoresinol

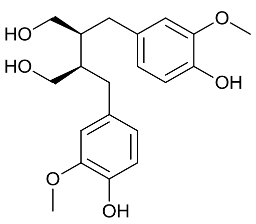

(+)-secolariciresinol

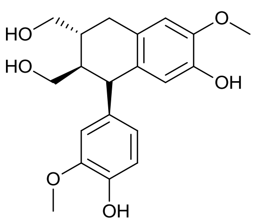

(+)-isolariciresinol

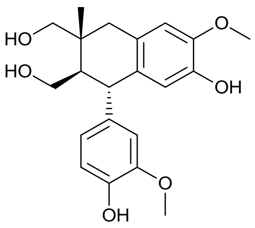

cycloolivil
